# A deep intronic *CLRN1* (*USH3A*) founder mutation generates an aberrant exon and underlies severe Usher syndrome on the Arabian Peninsula

**DOI:** 10.1038/s41598-017-01577-8

**Published:** 2017-05-03

**Authors:** Arif O. Khan, Elvir Becirovic, Christian Betz, Christine Neuhaus, Janine Altmüller, Lisa Maria Riedmayr, Susanne Motameny, Gudrun Nürnberg, Peter Nürnberg, Hanno J. Bolz

**Affiliations:** 1 Eye Institute, Cleveland Clinic Abu Dhabi, Abu Dhabi, United Arab Emirates; 2 Division of Pediatric Ophthalmology, King Khaled Eye Specialist Hospital, Riyadh, Saudi Arabia; 3 Department of Pharmacy - Center for Drug Research, Ludwig-Maximilians-Universität München, München, Germany; 4 Bioscientia Center for Human Genetics, Ingelheim, Germany; 5 Cologne Center for Genomics (CCG), University of Cologne, Cologne, Germany; 6 Institute of Human Genetics, University Hospital of Cologne, Cologne, Germany; 7 Cologne Excellence Cluster on Cellular Stress Responses in Aging-Associated Diseases (CECAD), University of Cologne, Cologne, Germany; 8 Center for Molecular Medicine Cologne (CMMC), University of Cologne, Cologne, Germany

## Abstract

Deafblindness is mostly due to Usher syndrome caused by recessive mutations in the known genes. Mutation-negative patients therefore either have distinct diseases, mutations in yet unknown Usher genes or in extra-exonic parts of the known genes – to date a largely unexplored possibility. In a consanguineous Saudi family segregating Usher syndrome type 1 (USH1), NGS of genes for Usher syndrome, deafness and retinal dystrophy and subsequent whole-exome sequencing each failed to identify a mutation. Genome-wide linkage analysis revealed two small candidate regions on chromosome 3, one containing the *USH3A* gene *CLRN1*, which has never been associated with Usher syndrome in Saudi Arabia. Whole-genome sequencing (WGS) identified a homozygous deep intronic mutation, c.254–649T > G, predicted to generate a novel donor splice site. *CLRN1* minigene-based analysis confirmed the splicing of an aberrant exon due to usage of this novel motif, resulting in a frameshift and a premature termination codon. We identified this mutation in an additional two of seven unrelated mutation-negative Saudi USH1 patients. Locus-specific markers indicated that c.254–649T > G_*CLRN1*_ represents a founder allele that may significantly contribute to deafblindness in this population. Our finding underlines the potential of WGS to uncover atypically localized, hidden mutations in patients who lack exonic mutations in the known disease genes.

## Introduction

Usher syndrome is the most common cause of inherited deafblindness^1^. Type 1 (USH1) is characterized by congenital deafness and early (first decade) retinitis pigmentosa (RP), whereas type 2 (USH2) displays progressive hearing impairment and RP of later onset. USH3 is characterized by progressive hearing loss, RP, and variable peripheral vestibular dysfunction^2^. However, disease resulting from mutations in the *USH3A* gene, *CLRN1*, is variable, ranging from non-syndromic RP^3^ to USH1^4^. The advent of next-generation sequencing (NGS) has enabled panel-sequencing of the 11 known Usher genes, and its application in a recent study on European deafblind patients identified the causative mutations in the majority^5^. In a Saudi Arabian family with four siblings affected by Usher syndrome type 1, escalating the genetic investigations from gene panel NGS over genome-wide linkage analysis to whole-exome sequencing (WES) and finally whole-genome sequencing (WGS) led up to the molecular diagnosis. Our study demonstrates the potential of WGS to unlock hidden mutations.

## Results

### NGS of gene panels for retinal dystrophy and for deafness

Apart from a heterozygous frameshift mutation in *TUBGCP6*, c.5001_5003delinsCA (p.Gln1667Hisfs*11), NGS of the known genes for Usher syndrome, for other syndromic and isolated hearing loss, and for retinal degeneration did not identify any mutations. Biallelic *TUBGCP6* mutations cause microcephalic primordial dwarfism and additional congenital anomalies, including retinopathy^6^. Given the recessive inheritance and additional symptoms associated with mutations in *TUBGCP6* (which are not present in the affected family members analyzed in our study), the apparently monoallelic variant most likely represents carriership for an unrelated disorder. Our results from NGS panel analysis thus largely excluded not only mutations in the coding sequences of the Usher syndrome genes and genes causing similar syndromes (e.g. USH3-like PHARC due to *ABHD12* mutations^7^), but also simultaneous mutations in a deafness gene and an RP gene mimicking Usher syndrome. Quantitative analysis of NGS reads did not indicate large copy number variations (CNVs) such as deletions of one or several contiguous exons.

### Whole-exome sequencing (WES)

**Figure 1:**
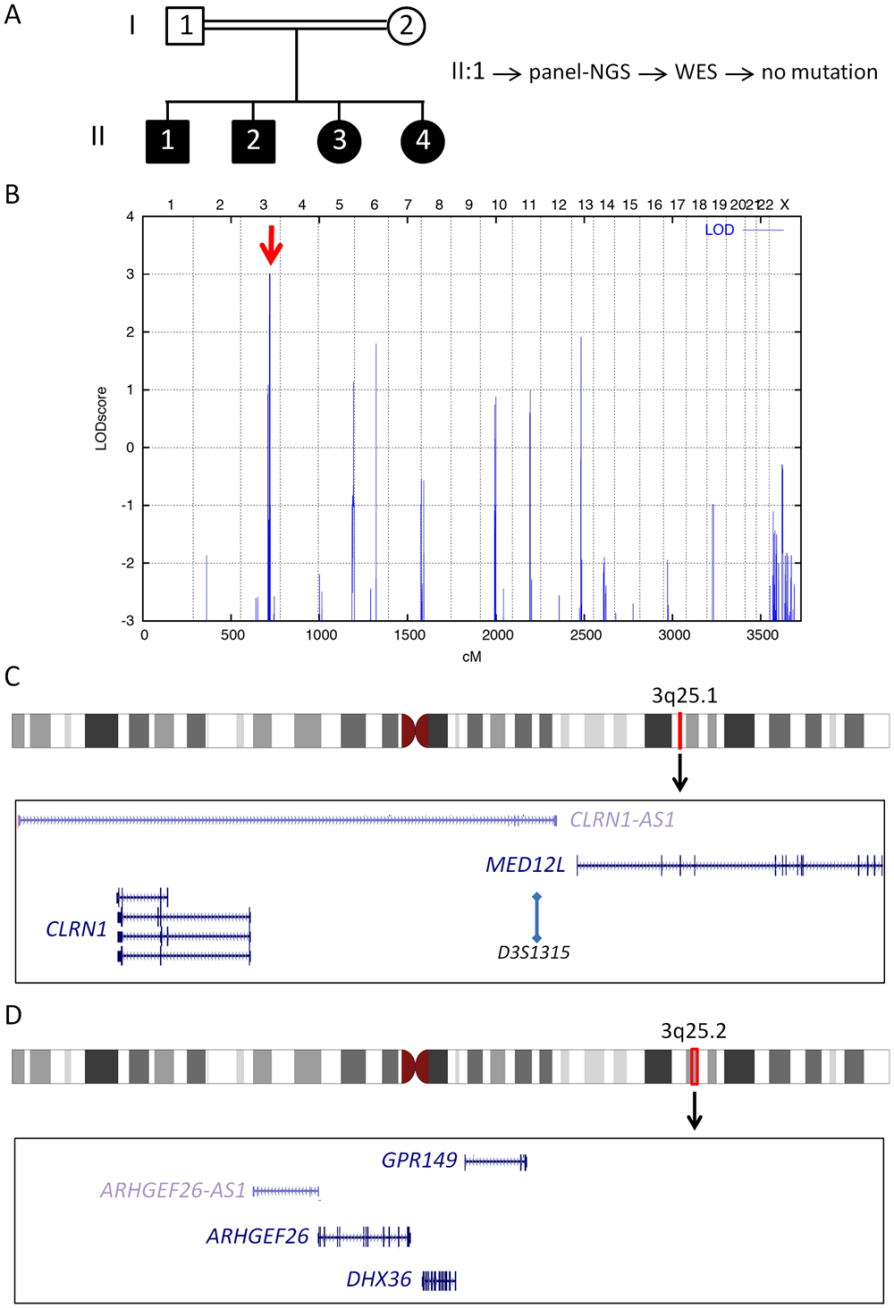
(**A**) Pedigree of the Saudi family with Usher syndrome type 1. Parents are distantly related. The sample of patient II:1 were subjected to NGS of an Usher gene panel and subsequently to WES – without finding a mutation. (**B**) Samples of the parents and the four affected children were subjected to genome-wide linkage analysis. The graphical view of LOD score calculation illustrates a combined maximum parametric LOD score of 3.01 for two neighboring regions on chromosome 3. (**C**) The candidate interval on chromosome 3q25.1 comprises the *CLRN1* gene (the position of the polymorphic microsatellite marker *D3S1315* is indicated; the other microsatellite markers that have been used for haplotyping flank this HBD region at the centromeric or telomeric side), whereas (**D**) the 3q25.2 region does not contain a known Usher gene. *CLRN1-AS1*, *CLRN1* antisense RNA 1; *MED12L*, Mediator of RNA polymerase II transcription subunit 12-like protein; *ARHGEF26*, Rho guanine nucleotide exchange factor 26; *ARHGEF26-AS1*, *ARHGEF26* antisense RNA 1; *DHX36*, DEAH-box helicase 36; *GPR149*, G protein-coupled receptor 149.

WES data were filtered for rare homozygous variants (see Methods), which revealed 38 such variants in 37 genes. As could be expected after mutation-negative panel-NGS, none of these variants affected a gene implicated in Usher syndrome, RP, or recessive deafness. The family structure with distant parental consanguinity and four affected siblings was highly suitable for an efficient linkage analysis. Hence, to identify the causative mutation, we set out to apply this approach (Fig. 1A).

### Genome-wide linkage analysis

Compatible with the distant consanguinity of the parents, we identified only two neighboring regions with homozygosity by descent (HBD) of very small size and a combined maximum parametric LOD score of 3.01 (Fig. 1B) on chromosome 3q25.1 (150,609,866–150,911,683; 301.817 kb; Fig. 1C) and 3q25.2 (153,396,096–154,676,122; 1.28 Mb; Fig. 1D). These regions contained two and three annotated genes, respectively. Of note, the *USH3A* gene, *CLRN1*, was contained in the 3q25.1 region. None of the 38 homozygous variants identified by WES located in the two neighboring candidate regions on chromosome 3.

### Whole-genome sequencing (WGS)

Given the results of panel-NGS, WES and genome-wide linkage analysis, we hypothesized that the causative mutation was likely to reside in a non-coding region, and possibly within the *CLRN1* gene. Of note, and in line with the results from genome-wide linkage analysis, filtering of WGS data for rare homozygous variants (see Methods) identified only one such variant, located within one of the two mapped adjacent HBD regions on chromosome 3: g.150660197A > C (c.254–649T > G) in *CLRN1*. Because the c.254–649T > G_*CLRN1*_ affects an intron (between “exon 0b” – an exon contained in transcript NM_001256819.1 – and exon 1) of a proven Usher syndrome gene, and because *in silico* analysis predicted aberrant splicing (see below), we focused on this alteration. Moreover, it has not been annotated in the 1000 Genomes Project. Compatible with its deep intronic location, the variant is absent from exonic sequence databases. It has not been reported previously and is therefore also absent from the HGMD.

### Minigene splice assay

**Figure 2:**
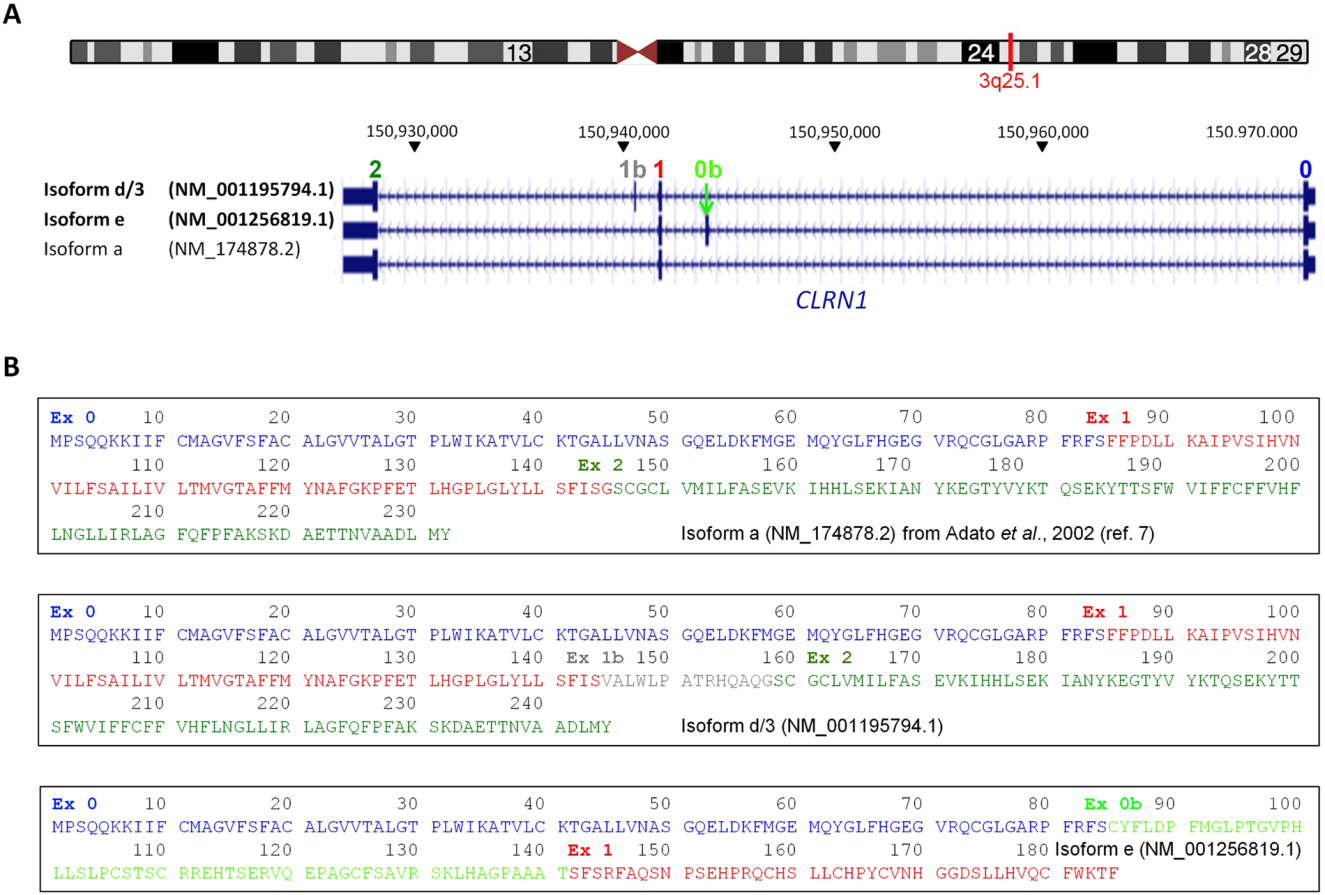
Scheme of UCSC-annotated Refseq *CLRN1* isoforms (hg38). (**A**) For our construct, we refer to a combination of isoforms d/3 (NM_001195794.1) and e (NM_001256819.1) that also includes the exons of the “major isoform”, isoform a (NM_174878.2)^8^: exons 0, 1 and 2. We designated additional exons, which are located between exons 0 and exon 1 and between exons 1 and exon 2, respectively, as exon 0b and exon 1b. (**B**) Protein sequences of the three above isoforms. In NM_001256819.1, the inclusion of exon 0b shifts the reading frame of exon 1, resulting in a different peptide sequence for exon 1 in this isoform. Note that, in contrast to the scheme in Fig. 3, the isoforms are in antisense orientation.

**Figure 3:**
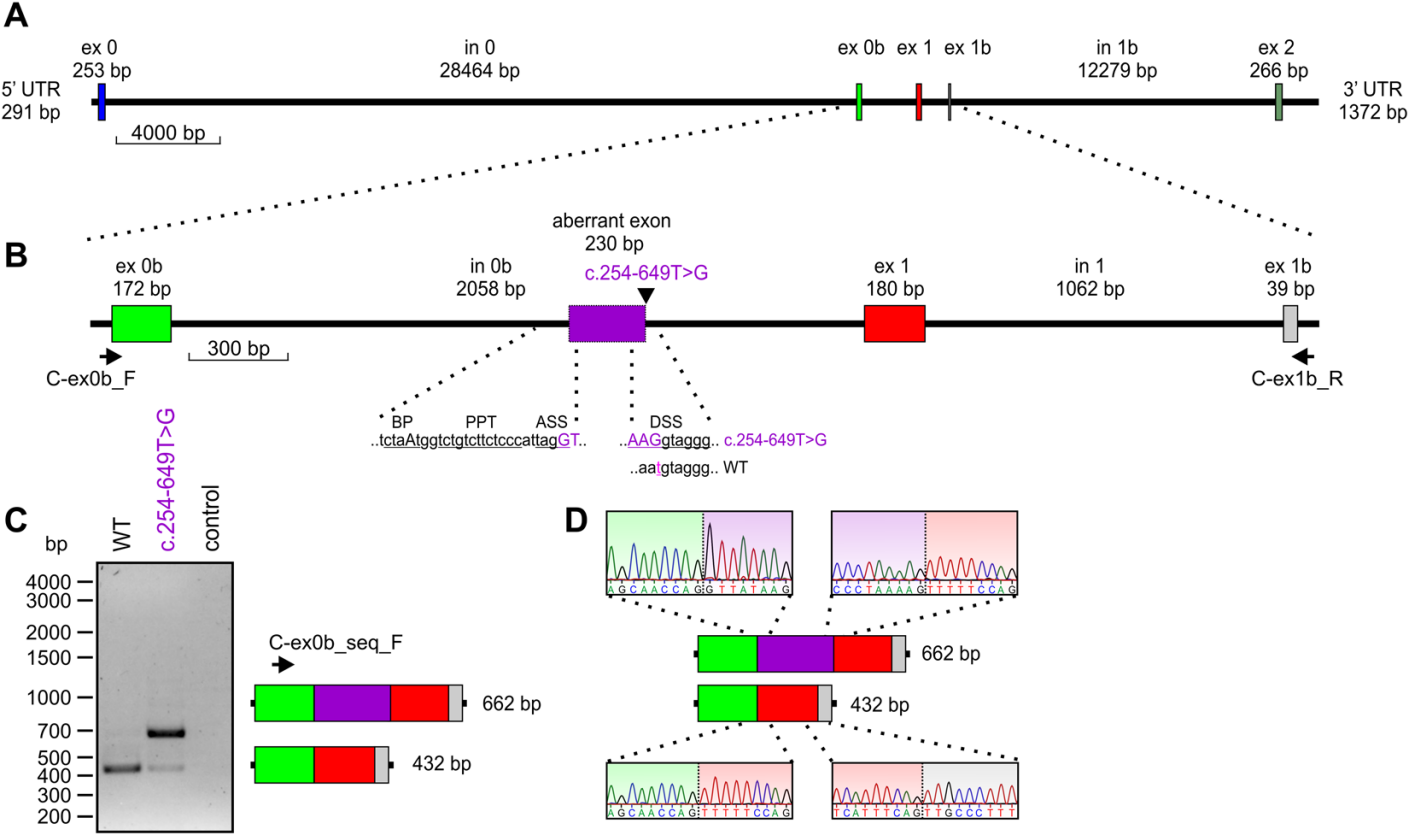
*CLRN1* minigene splice assay. (**A**) Schematic to scale overview of the genomic *CLRN1* structure including the 5′ and 3′ untranslated regions (5′ UTR and 3′ UTR, respectively). bp, basepairs. Exons (ex) are shown as colored boxes. (**B**) *CLRN1* to scale minigene used for the splice experiments shown in (**C**,**D**). The position of the c.254–649 T > G mutation is indicated by an arrowhead and the novel exon generated by the mutation (referred to as “aberrant exon”) is displayed as a dashed purple box. The dashed lines represent a schematic magnification of the 3′ and 5′ splice sites flanking the aberrant exon. The consensus sequences for the putative branch point (BP), the polypyrimidine tract (PPT), the 3′ acceptor splice site (ASS), and the 5′ donor splice site (DSS) of the aberrant exon are underlined. The putative lariat-forming adenosine in the PPT is shown in upper case. The binding positions of the primers used for the RT-PCR shown in (**C**) are indicated by arrows. (**C**) Representative RT-PCR analysis from HEK293 cells transiently transfected with the single constructs as indicated. As control, RT-PCR from non-transfected HEK293 cells was performed. The length and exon composition of the two splice products for WT (432 bp) and the c.254–649T > G mutant (432 bp and 662 bp) are shown on the right panel. The position of the primer used for Sanger sequencing shown in (**D**) is indicated by an arrow. (**D**) Representative electropherograms of the exon-exon boundaries for both RT-PCR products of the c.254–649T > G mutant.

**Figure 4:**
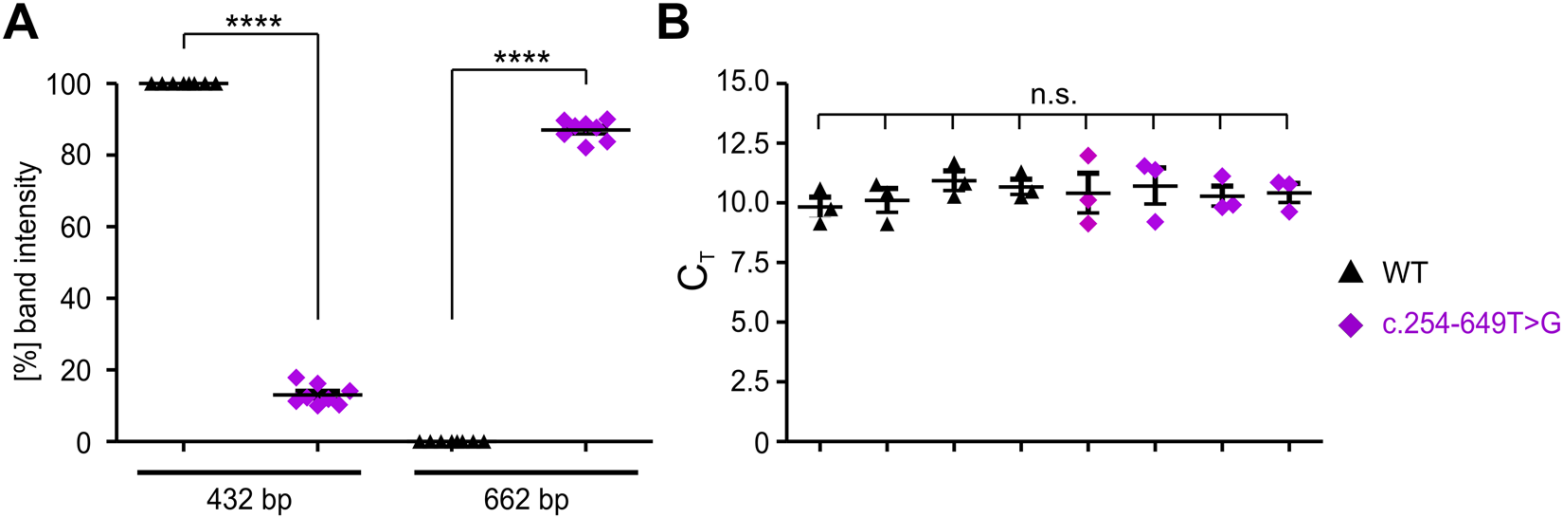
Quantification of the RT-PCR results shown in Fig. 3C. (**A**) Quantification of eight RT-PCR experiments resulting from four independent transfection experiments for both WT and c.254–649T > G mutant for the 432 bp and 662 bp bands. The single values are given as percentages of the total band intensities ± standard error of the mean (SEM). (**B**) Quantification of the single WT and the c.254–649T > G cDNAs used for the calculation of band intensities displayed in (**A**). Shown are the mean C_T_ values (n = 3) ± SEM for aminolevulinic acid synthase (ALAS) as housekeeper gene. The single values are summarized in Table 1. For statistical analysis, one-way ANOVA followed by Tukeys test was used. *p < 0.05; **p < 0.01; ***p < 0.001; ****p < 0.0001.

*In silico* analysis of the c.254–649T > G mutation using Spliceview and Maximum Entropy predicts that the mutation generates a novel donor splice site (score of 85 [Spliceview] and 8.76 [Maximum Entropy model]) compared to no predicted donor site in the wild-type sequence). Several potential acceptor sites are predicted in the wild-type sequence 5′ of the alteration. Because we could not detect *CLRN1* in RT-PCR analysis from whole blood of the patients, we established a minigene-based assay suitable for analysis in commonly used human cell lines. Due to the genomic dimension of *CLRN1* (>46 kb, Figs 2 and 3A), minigene-based analysis of *CLRN1* mRNA splicing could not be examined with a construct comprising all five exons (exons 0, 0b, 1, 1b, 2) and introns. Hence, we designed a *CLRN1* minigene of convenient size encompassing approx. 3.6 kb and harboring three annotated *CLRN1* exons and the interjacent native introns (Fig. 3B). According to the established nomenclature of the major *CLRN1* transcripts^8^, the respective exons were termed exon 0b, exon 1 and exon 1b (Fig. 3A,B). The minigenes containing the healthy (herein referred to as wild-type, WT) and the mutant variant were transiently transfected to HEK293 cells. In subsequent RT-PCR analysis for the WT *CLRN1* minigene, we exclusively detected the correctly spliced transcript, validating the suitability of the minigene assay in this cell line. Splicing of the mutant construct with the c.254–649T > G mutation resulted in an additional band besides the correctly spliced band, indicating aberrant splicing (Fig. 3C,D). Subsequent semi-quantitative analysis of the band intensities for the c.254–649T > G mutation revealed that, compared to the correctly spliced variant, this aberrant splice product is predominant (87% *versus* 13%, Fig. 4). Sequencing of the band corresponding to the novel splice variant showed that the c.254–649T > G mutation generates an aberrant exon in intron 0b. This aberrant exon comprises 230 bp (Fig. 3B,E). If included into the major *CLRN1* isoform (isoform a, NM_174878.2), the aberrant exon leads to a frameshift and a premature stop codon, predicting either a truncated protein (106 residues compared to 232 residues of the NM_174878.2-deduced wild-type protein, with the inclusion of 22 unrelated residues) or an unstable transcript subjected to nonsense-mediated decay (NMD). However, irrespective of the *CLRN1* splice isoform, the insertion of the aberrant exon is expected to result in profound alteration or complete deficiency of CLRN1 protein. According to the ACMG guidelines, the c.254–649T > G variant can be claimed as pathogenic (Table 3 and Table 5 in Richardson *et al*.^9^), with the following classification criteria for pathogenic variants applying here: (i) 1 Very strong (PVS1 null variant: canonical splice site (being generated by the mutation) in a gene where loss-of-function is a known mechanism of disease) AND (a) ≥1 Strong (PS3: functional studies supportive of a damaging effect, PS4: observation of the variant in multiple unrelated patients with the same phenotype). In addition, the variant is absent from controls (Moderate, PM2).

**Table 1:** Mean and SEM values of the data displayed in Fig. 4.

	Percentage of band intensity				CT values (ALAS) for single cDNAs							
	432 bp		662 bp									
	WT	c.254–649T > G	WT	c.254–649T > G	WT				c.254–649T > G			
M_V_	100,0	13,0	0,0	87,0	9,83	10,11	10,93	10,67	10,41	10,71	10,29	10,42
SEM	0,0	1,0	0,0	1,0	0,42	0,50	0,41	0,32	0,83	0,76	0,42	0,40

### Targeted mutation analysis in NGS-panel-negative Saudi Arabian USH patients and haplotype analysis

**Figure 5:**
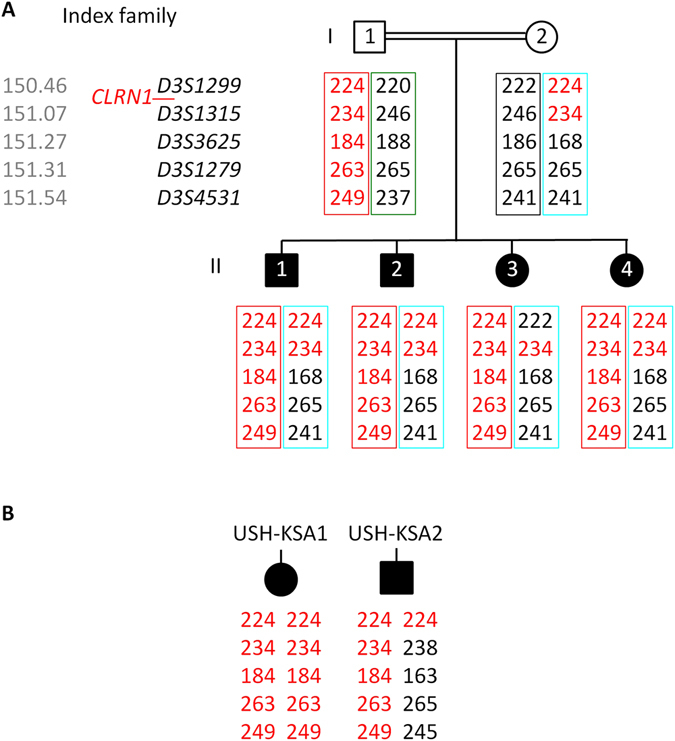
Genotyping of polymorphic microsatellite markers from the *CLRN1* (*USH3A*) locus on chromosome 3q25.1 in (**A**) the index family and (**B**) the two additional Saudi patients (USH-KSA1 and USH-KSA2) who also carry the c.254–649T > G_*CLRN1*_ mutation in homozygous state. Grey numbers indicate the marker positions in Mb on chromosome 3 (according to hg38). Numbers indicate the PCR product size obtained with the primer pairs given in the UCSC Genome Browser. Only *D3S1315* localizes within one of the two HBD regions that has been mapped in the index family. *D3S1299* is located centromeric of that HBD region while *D3S3625*, *D3S1279* and *D3S4531* are telomeric of that region. The putative original c.254–649T > G-associated haplotype (red) is preserved on the paternal allele in the index family (paternal and maternal haplotypes are indicated by boxes of different colors), on one allele of patient USH-KSA2, and on both alleles of patient USH-KSA1.

We identified the c.254–649T > G_*CLRN1*_ mutation in homozygous state in two (here referred to as USH-KSA1 and USH-KSA2) out of seven additional patients with Usher syndrome type 1 from Saudi Arabia in whom targeted NGS covering all coding exons of the known Usher syndrome genes had not identified any mutation. Genotyping of locus-specific microsatellite markers (*D3S1299*, *D3S1315*, *D3S3625*, *D3S1279* and *D3S4531*, spanning about 1 Mb) in all members of the index family and both additional patients, USH-KSA1 and USH-KSA2, revealed a disease-associated haplotype which was preserved on the paternal allele in the index family and in patients USH-KSA1 and USH-KSA2 over the whole range covered by the above markers, indicating that these patients have a common ancestor who carried the mutation (Fig. 5).

## Discussion

About 85% of the mutations underlying Mendelian traits localize in the protein-coding exons of these genes, or they affect the splice sites adjacent to the coding sequences^10^. For phenotypes like Usher syndrome, with mutations in the known genes explaining the vast majority of cases, targeted NGS of panels comprising these genes are highly effective in confirming the clinical diagnosis. The determination of the causative mutations is important for personalized management of patients because it enables clinical prognoses (differentiation of clinical subtypes), in particular in hearing-impaired children before onset of retinal degeneration. Although combined impairment of hearing and vision, commonly termed deafblindness, is due to Usher syndrome in most cases, other conditions should be excluded, amongst other reasons because they may be treatable (e.g. Refsum syndrome that may respond to phytanic acid-reduced diet^11^).

The phenotype in the family reported herein is clearly inherited (parental consanguinity, four affected siblings) and compatible with Usher syndrome. Lack of mutations in the exons of the genes known to cause Usher syndrome (and clinically overlapping conditions) may indicate rare atypically localized mutations, outside the protein-coding exons. Such mutations have been reported for different retinopathies including Usher syndrome: They may affect non-coding exons and possibly affect gene transcription, as we and others have shown for *EYS*-related RP^12^. Deep intronic mutations, as in case of *OFD1*-associated RP^13^, the prevalent c.2991 + 1655A > G *CEP290* mutation in LCA^14^, or certain *USH2A* mutations in USH2^15, 16^) have been shown to generate aberrant exons through missplicing. Moreover, mutations may reside outside genes. For example, structural variations and point mutations may disturb normal chromatin folding with consecutive gene misexpression, a disease mechanism known from developmental disorders and cancer^17^. Finally, the recent identification of mutations in *CEP78* in patients with an Usher-like phenotype^18^ illustrates that novel disease genes have to be taken into account even in mutation-negative Usher syndrome patients.

It has been estimated that about one third of disease-causing mutations may cause aberrant splicing. While splice mutations affecting splice site consensus sequences (some 10% of disease-causing mutations^19^) are easy to recognize, those in less conserved sequence motifs are more difficult, but, if exonic, are at least captured by Sanger, NGS-panel, or whole-exome sequencing. Because deep intronic splice site mutations escape detection by standard sequencing approaches, they represent the most challenging mutation of this category. Our finding highlights the diagnostic potential of whole-genome sequencing (WGS) in finding mutations in the 99% of the genome that are not protein-coding. WGS has been shown to be superior to WES in identifying disease-causing mutations, amongst other things because of more uniform coverage and its ability to detect structural genomic mutations^20, 21^. However, the need for extensive data storage and the high costs of WGS have so far impeded its routine diagnostic application. Moreover, a minigene assay was necessary in our study to prove pathogenicity of c.254–649T > G_*CLRN1*_. This would be impossible in a routine diagnostic setting and demonstrates that final interpretation of deep intronic variants suspected to cause aberrant splicing will remain challenging. Furthermore, in our minigene assay the investigated exons are not in their native genomic and cellular environment. We assume that c.254–649T > G-associated missplicing is very likely to occur in retinal and cochlear cells and in the way we describe here, but this cannot be finally proven by our data.

With only 30 supposedly pathogenic variants annotated in the Human Gene Mutation Database^22^, the *CLRN1*-associated subtype of Usher syndrome, *USH3A*, is very rare. However, due to founder mutations, it represents the predominant subtype in Finland^23^ and in some Jewish populations^2^. To our knowledge, our findings represent the first description of *USH3A* in the Saudi Arabian population. Its hidden localization has prevented its identification so far. The presence of c.254–649T > G_*CLRN1*_ in three Saudi USH1 patients and a mutation-associated haplotype (spanning at least 1 Mb) indicate a founder mutation that may significantly contribute to Usher syndrome in this population. We therefore recommend to consider this mutation in genetic analysis of patients with all clinical subtypes, explicitly including USH1 (all patients in our study were diagnosed as USH1). Because therapeutic strategies for *USH3A* are being developed^24^, pinpointing the molecular diagnosis may become crucial for *USH3A* patients’ medical care in the future. As a future treatment strategy to eliminate the abnormal splicing due to c.254–649T > G_*CLRN1*_, CRISPR/Cas9-based genome editing may become a promising approach for patients with this mutation. Because modifications of deep intronic regions do not affect the coding sequence of the respective gene, this technology seems predestined for treatment of disease-associated mutations in these regions.

## Methods

All methods were carried out in accordance with the approved guidelines.

### Patients

The study was approved by the institutional review boards of the Ethics Committee of the University Hospital of Cologne and the King Khaled Eye Specialist Hospital, Riyadh. Informed consent for genetic investigations was obtained from the parents. Clinical and specimen investigations were conducted according to the Declaration of Helsinki.

### NGS of gene panels for inherited retinal dystrophies and deafness

The coding exons of 11 Usher syndrome genes (*MYO7A/USH1B*, *USH1C*, *CDH23/USH1D*, *PCDH15/USH1F*, *USH1G*, *USH2A*, *DFNB31/USH2D*, *GPR98/USH2C*, *CLRN1/USH3A*, *PDZD7/*digenic/*USH2A*-modifier, *CIB2*; 398 exons) and 17 genes whose mutations underlie conditions clinically similar to Usher syndrome (*CEP250*, *HARS*, *ABHD12*, *PEX1*, *PEX2*, *PEX3*, *PEX5*, *PEX6*, *PEX7*, *PEX10*, *PEX12*, *PEX13*, *PEX14*, *PEX16*, *PEX19*, *PEX26*, *PHYH*) were enriched using Roche/NimbleGen sequence capture technology, sequenced on an Illumina HiSeq 1500 system and bioinformatically evaluated as described previously^12^. Another gene whose biallelic mutations have very recently been reported to cause Usher syndrome, *CEP78* ^18^, was not yet included in our panel. However, because patients with *CEP78* mutations appear to have cone-rod dystrophy rather than RP^25, 26^, we would not consider *CEP78* a *bona fide* Usher gene. Quantitative readout of NGS reads to exclude CNVs was carried out as described previously^12^. Besides the explicitely mentioned genes above, the used NGS panels contain virtually all genes known to be involved in non-syndromic and syndromic forms of hearing loss (n = 119; Suppl. Table 1) and retinal degeneration (n = 155; Suppl. Table 2), respectively, at the time of panel design (2015). These genes were enriched and sequenced in parallel (with very little redundancy: a few genes, like *ABHD12*, *CLRN1* and *USH1C* are present on both panels because their mutations may cause either syndromic hearing loss or non-syndromic RP). The bioinformatic pipeline was consulted for putatively pathogenic variants not only in Usher syndrome genes, but also in these genes.

### Genome-wide linkage analysis

DNA was extracted from peripheral blood samples using standard methods. DNA samples of the parents and the four affected siblings (family as displayed in Fig. 1A) were analyzed for genome wide linkage using the Infinium CoreExome-24 v1.1 BeadChip (Illumina Inc., San Diego, CA) according to the manufacturer’s protocol. Subsequent data handling was performed using the graphical user interface ALOHOMORA^27^. Relationship errors were identified by using the program Graphical Relationship Representation^28^. The program PedCheck was applied to find Mendelian errors^29^ and data for SNPs with such errors were removed from the data set. Non-Mendelian errors were identified by using the program MERLIN^30^ and unlikely genotypes for related samples were deleted. Linkage analysis was performed assuming autosomal-recessive inheritance, full penetrance, consanguinity, and a disease gene frequency of 0.0001. Multipoint LOD scores were calculated using the program Allegro^31^. Haplotypes were reconstructed with Allegro and presented graphically with HaploPainter^32^. Regions of homozygosity by descent (HBD) were annotated with their positions corresponding to NCBI Build 37.

### Whole-exome sequencing

Genomic DNA of patient II:1 (Fig. 1A) was subjected to whole-exome sequencing, WES. Exome capture was performed using the Agilent SureSelectXT Human All Exon 50 Mb kit following manufacturer’s procedures (Agilent, Santa Clara, CA, USA) and sequenced with Illumina paired end sequencing (protocol v1.2). Briefly, DNA was sheared by fragmentation (Covaris, Woburn, MA, USA) and purified using Agencourt AMPure XP beads (Beckman Coulter, Fullerton, CA, USA). Resulting fragments were analysed using an Agilent 2100 Bioanalyzer. Fragment ends were repaired and adaptors were ligated to the fragments. The library was purified using Agencourt AMPure XP beads and amplified by PCR before hybridisation with biotinylated RNA baits. Bound genomic DNA was purified with streptavidin coated magnetic Dynabeads (Invitrogen, Carlsbad, CA, USA) and re-amplified to include barcoding tags before pooling for sequencing on an paired-end, 100 cycle run on an Illumina HiSeq 2000 according to manufacturer’s protocols. Briefly, primary data were filtered according to signal purity by the Illumina Realtime Analysis (RTA) software v1.8. Subsequently, reads were mapped to the human genome reference build hg19 using the bwa-aln^33^ alignment algorithm. GATK v1.6^34^ was used to mark duplicated reads, for local realignment around short insertions and deletions, to recalibrate the base quality scores and to call SNPs (incorporating variant quality score recalibration) and short indels^35^. Scripts developed in-house at the Cologne Center for Genomics were used to detect protein changes, affected donor and acceptor splice sites, and overlaps with known variants. Analysis for acceptor and donor splice site mutations and for the activation of new aberrant splice sites was carried out with a Maximum Entropy model^36^ and filtered for effect changes. In particular, and because the patients came from a consanguineous background, we filtered for high-quality (coverage > 15; quality > 25) rare (MAF < 0.005) homozygous variants (dbSNP build 135, the database of the 1000 Genomes Project build 20110521, TGP^37^), and the Exome Variant Server, NHLBI Exome Sequencing Project, Seattle, build ESP6500^38^). We also filtered against an in-house database containing all variants from 511 exomes from epilepsy patients to exclude pipeline-related artifacts/false positives (MAF < 0.004). In addition to the above large-scale sequencing databases consulted, a local pipeline^35^ and interface was used (Varbank v.2.3; https://varbank.ccg.uni-koeln.de) as described previously^39, 40^, and we searched the Exome Aggregation Consortium (ExAC) database^41^ (as of 05/2016), which aggregates numerous databases including the current versions of the ESP and the TGP, for homozygous candidate variants from the mapped regions.

### Whole-genome sequencing

The library was prepared and size selected by using the Illumina^®^ TruSeq^®^ DNA Sample Preparation Kit and Agencourt AMPure XP beads, starting with 1,2 µg genomic DNA and followed by one cycle of PCR to complete adapter structure. The library was validated with the Agilent 2200 TapeStation and quantified by qPCR. Using an Illumina HiSeq X Ten Sequencer, we generated 423M 150-bp paired-end reads corresponding to 126,75 Gb of sequence data and an average coverage of 39-fold.

### Bioinformatic analysis of WGS data

845,688,028 150 bp paired-end reads were generated from sequencing. They were mapped to the hg19 reference genome using BWA-ALN^33^ (version 0.6.2). After mapping, duplicates were marked using Picard (version 1.64; http://picard.sourceforge.net) and basecalling quality score recalibration and local indel realignment was performed using GATK^34^ (version 1.6.11). Enrichment statistics computed by Picard on the resulting BAM file showed a sufficient and rather uniform coverage of the 1.6 Mb target region (mean coverage 39×, 87.6% of target covered by at least 30×, 98.9% of target covered by at least 20×, 99.8% of target covered by at least 10×). Variants were called genome wide using samtools mpileup^42^ (version 0.1.18) and in the complete target region using GATK UnifiedGenotyper (version 1.6.11). The resulting variants were annotated with software developed at the CCG based on the ENSEMBL b68 gene models and filtered to exclude variants of low confidence (alternative allele frequency <10%, number of reads at variant position <5, variant quality score <10, number of reads supporting the variant <3). The remaining variants were annotated with their presence in public databases (dbSNP^43^, 1000 Genomes Project^44^, Exome Variant Server (EVS http://evs.gs.washington.edu/EVS/), dbVAR and DGVa^45^, GERP^46^, ENSEMBL^47^, and the commercial HGMD professional database^48^) as well as a CCG inhouse exome collection of 511 samples. Effects on splicing were predicted using the maximum entropy approach from Yeo and Burge^36^ and SIFT^49^, POLYPHEN^50^, and RVIS^51^ scores for all coding variants were taken into account. The GATK UnifiedGenotyper variant list was used to compute regions of homozygosity with Allegro^31^. The annotated variant lists were uploaded to the CCG’s varbank (https://varbank.ccg.uni-koeln.de) database for further evaluation.

### Sanger sequencing

Validation of the *CLRN1* candidate variant c.254–649T > G, segregation analysis and screening of so far NGS-panel-negative Saudi Arabian Usher syndrome patients for this mutation were carried out by Sanger sequencing. For this, we PCR-amplified a 571 bp fragment comprising the position of the mutation, using the forward primer *CLRN1*-mF: 5′-ggttataagctctgtgagacaac-3′ and the reverse primer *CLRN1*-mR: 5′-ccaagcctttaatgacctttctcg-3′. PCR amplification was carried out on a Biometra T3000 PCR cycler (Analytik Jena, Jena, Germany) as follows: 1× (95 °C, 15 min), 15× (95 °C, 1 min./68 °C (reduced by 0.5 °C in every subsequent cycle), 1 min./72 °C, 1 min.), 30× (95 °C, 1 min./60 °C, 1 min./72 °C, 1 min.), 1× 72 °C, 10 min.

### Minigene splice assay

Attempts to investigate *CLRN1* splicing via cDNA amplification and sequencing based on RNA isolated from whole blood of the patients were not successful. We thus chose a splicing minigene splice assay based on the *CLRN1* genomic sequence (Figs 2 and 3A). Several *CLRN1* isoforms have been annotated, and apart from the three protein-coding exons 0, 1 and 2 of isoform a (NM_174878.2), the major isoform^8^, there was no consistent numbering of exons available. Our investigation of splicing was based on two transcript isoforms which include additional exons between exon 0 and exon 1 (in isoform e, NM_001256819.1) and between exons 1 and 2 (isoform d, NM_001195794.1) (Fig. 3B). For maintaining compatibility with exon numbering of isoform a^8^, we designated these additional exons as exon 0b and 1b, respectively (Fig. 3A,B). The WT and mutant minigenes (3,552 bp each) were synthesized by BioCat (Heidelberg, Germany) and delivered in the pcDNA3.1 eGFP standard vector. For RT-PCR analysis, HEK293 cells were transiently transfected using the CaPO3 method. 24 h post transfection, cells were harvested and the RNA was isolated using the RNeasy Mini Kit (QIAGEN, Hilden, Germany) according to the manufacturer’s instructions. Subsequent cDNA synthesis was conducted with equal amounts of RNA (1 µg each) using the RevertAid First Strand cDNA Synthesis Kit (Thermo Scientific). For subsequent PCR, following primer were used: C-ex0b_F 5′- ctatcttgttgttgatgcaggc-3′ and C-ex2_R 5′-gtgtcaagagcaagaaagtacc-3′. The single PCR products representing the WT and the c.254–649T > G mutant splice isoforms were extracted, purified, and sequenced. Sequencing was conducted by Eurofins Genomics (Ebersberg, Germany) using the following primer: C-ex0b_seq_F 5′-ccttcatgggactcccaacag-3′. Semi-quantitative analysis of the band intensities from electrophoresis on an agarose gel was performed on eight technical replicates resulting from four different transfections. For this purpose, the single sets of two RT-PCR experiments for the WT and the c.254–649T > G mutant were conducted with a variable number of cycles ranging between 25–30. PCR was performed with the Herculase II Fusion DNA Polymerase (Agilent Genomics) using the following conditions: 1× (95 °C, 2 min), 25–30× (95 °C, 20 sec; 60 °C 20 sec; 72 °C, 2 min), 1× (72 °C, 5 min). The absolute intensities of the single PCR bands were calculated by the Image Lab software (BioRad, Hercules, CA, U.S.A.). The single cDNAs resulting from the four independent transfections for WT and for the c.254–649T > G mutant were quantified by a StepOnePlus Real-Time PCR System (Applied Biosystems) using SYBR Select Master Mix (Applied Biosystems). The following primers specific for human aminolevulinic acid synthase (ALAS) as a housekeeper gene were used: ALAS fwd: 5′-GATGTCAGCCACCTCAGAGAAC-3′ and ALAS rev: 5′-CATCCACGAAGGTGATTGCTCC-3′. For quantification, three technical replicates for each cDNA were conducted and the statistical comparison between the groups was done with one-way ANOVA, followed by the Tukey’s test for multiple comparisons. p < 0.05 was considered statistically significant.

### Characterization of the haplotype associated with the c.254–649T > G mutation in *CLRN1*

Genotyping of locus-specific microsatellite markers (*D3S1299*, *D3S1315*, *D3S3625*, *D3S1279* and *D3S4531*) was carried out in all members of the index family and in patients USH-KSA1 and USH-KSA2, using primers as given in the respective entries of the UCSC Genome Browser. For marker amplification, we applied the tailed primer method as described previously^52^. The forward primer of each marker was extended with a “tail” sequence 5′-TACGCATCCCAGTTTGAGACG-3′, and a FAM-labeled oligonucleotide complementary to this tail was added to the PCR reaction. The lengths of the PCR products (generated with Qiagen Hotstar Taq Polymerase) were determined by electrophoresis on an ABI-377 DNA sequencer. Genotypes were determined by GeneMapper (Applied Biosystems). PCR amplification of all markers was carried out as follows: 1× (95 °C, 15 min), 10× (95 °C, 30 sec./60 °C (reduced by 0.5 °C in every subsequent cycle), 40 sec./72 °C, 45 sec.), 25× (95 °C, 30 sec./57 °C, 40 sec./72 °C, 45 sec.), 1× 72 °C, 20 min.

## Electronic supplementary material

Supplementary Information
