# Supplementary material for: A deep intronic CLRN1 (USH3A) founder mutation generates an aberrant exon and underlies severe Usher syndrome on the Arabian Peninsula
Source: Sci Rep. 2017 May 3;7:1411. doi: 10.1038/s41598-017-01577-8 (PMC5431179; doi:10.1038/s41598-017-01577-8)
Supplement: Supplementary file 1 — Supplementary Information [file 41598_2017_1577_MOESM1_ESM.doc]

– Supplementary Information –

**A deep intronic *CLRN1* (*USH3A*) founder mutation generates an aberrant exon and underlies severe Usher syndrome on the Arabian Peninsula**

Arif O. Khan^1,2^*, Elvir Becirovic^3^*, Christian Betz^4^*, Christine Neuhaus^4^, Janine Altmüller^5,6^, Lisa Maria Riedmayr^3^, Susanne Motameny^5^, Gudrun Nürnberg^5^, Peter Nürnberg^5,7,8^, Hanno J. Bolz^4,6^

^1^Eye Institute, Cleveland Clinic Abu Dhabi, Abu Dhabi, United Arab Emirates

^2^Division of Pediatric Ophthalmology, King Khaled Eye Specialist Hospital, Riyadh, Saudi Arabia;

^3^Department of Pharmacy - Center for Drug Research, Ludwig-Maximilians-Universität München, München, Germany

^4^Bioscientia Center for Human Genetics, Ingelheim, Germany

^5^Cologne Center for Genomics (CCG), University of Cologne, Cologne, Germany

^6^Institute of Human Genetics, University Hospital of Cologne, Cologne, Germany

^7^Cologne Excellence Cluster on Cellular Stress Responses in Aging-Associated Diseases (CECAD), University of Cologne, Cologne, Germany

^8^Center for Molecular Medicine Cologne (CMMC), University of Cologne, Cologne, Germany

*These authors have contributed equally to the study.

Correspondence to: Arif O. Khan, [arif.khan@mssm.edu](mailto:arif.khan@mssm.edu) or Hanno J. Bolz, [hanno.bolz@uk-koeln.de](mailto:hanno.bolz@uk-koeln.de)

**TABLE S1**

**Deafness genes analyzed by NGS in this study.** ARNSHL, autosomal recessive non-syndromic hearing loss; ADNSHL, autosomal dominant non-syndromic hearing loss; XLNHSL, X-linked non-syndromic hearing loss; SHL, syndromic hearing loss. DFNA-, DFNB- and DFNX- designate loci for ADNSHL, ARNSHL and XLNSHL, respectively (and are therefore written in italics). "x", no locus designation. SANDD, sinoatrial node dysfunction and deafness; USH, Usher syndrome; PBD, peroxisome biogenesis disorder; PDS, Pendred syndrome; JLNS, Jervell and Lange-Nielsen syndrome; SeSAME, seizures, sensorineural deafness, ataxia, mental retardation and electrolyte imbalance; DDS, deafness dystonia syndrome. *STRC* could not be captured due to several homologous sequences in the genome.

| **Gene** | **ARNSHL** | **ADNSHL** | **XLNSHL** | **SHL** | **Modifier of** |
| --- | --- | --- | --- | --- | --- |
| *ABHD12* |  |  |  | "USH-like" (PHARC, see Table S2) |  |
| *ACTG1* |  | *DFNA20/26* |  |  |  |
| *ADCY1* | *DFNB44* |  |  |  |  |
| *ADGRV1* |  |  |  | *USH2C* |  |
| *ATP2B2* |  |  |  |  | *DFNB12, DFNA22* |
| *BDP1* | (x) |  |  |  |  |
| *BSND* | *DFNB73* |  |  |  |  |
| *CABP2* | *DFNB93* |  |  |  |  |
| *CACNA1D* |  |  |  | *SANDD* |  |
| *CEACAM16* |  | *DFNA4B* |  |  |  |
| *CEP250* |  |  |  | "USH-like" (with co-occurence of *C2orf71* mutation) |  |
| *CCDC50* |  | *DFNA44* |  |  |  |
| *CDH23* | *DFNB12* |  |  | *USH1D* |  |
| *CIB2* | *DFNB48* |  |  | *USH1J* |  |
| *CLDN14* | *DFNB29* |  |  |  |  |
| *CLIC5* | *DFNB103* |  |  |  |  |
| *CLRN1* |  |  |  | *USH3A* |  |
| *CLPP* | *DFNB81* |  |  | Perrault s. |  |
| *COCH* |  | *DFNA9* |  |  |  |
| *COL11A2* |  | *DFNA13* |  |  |  |
| *CRYM* |  | x |  |  |  |
| *DCDC2* | *DFNB66* |  |  |  |  |
| *DFNA5* |  | *DFNA5* |  |  |  |
| *DFNB31* | *DFNB31* |  |  | *USH2D* |  |
| *DFNB59* | *DFNB59* |  |  |  |  |
| *DIABLO* |  | *DFNA64* |  |  |  |
| *DIAPH1* |  | *DFNA1* |  |  |  |
| *DIAPH3* |  | *AUNA1* |  |  |  |
| *ELMOD3* | *DFNB88* |  |  |  |  |
| *EPS8* | *DFNB102* |  |  |  |  |
| *ESPN* | *DFNB36* | x |  |  |  |
| *ESRRB* | *DFNB35* |  |  |  |  |
| *EYA4* |  | *DFNA10* |  |  |  |
| *FAM65B* | *DFNB104* |  |  |  |  |
| *FOXI1* |  |  |  | *PDS* |  |
| *GIPC3* | *DFNB15/72/95* |  |  |  |  |
| *GJB2* | *DFNB1A* | *DFNA3A* |  |  |  |
| *GJB3* | x | *DFNA2B* |  |  |  |
| *GJB6* | *DFNB1B* | *DFNA3B* |  |  |  |
| *GPSM2* | *DFNB82* |  |  |  |  |
| *GRHL2* |  | *DFNA28* |  |  |  |
| *GRXCR1* | *DFNB25* |  |  |  |  |
| *GRXCR2* | *DFNB101* |  |  |  |  |
| *HARS* |  |  |  | "USH-like" |  |
| *HARS2* |  |  |  | Perrault s. |  |
| *HGF* | *DFNB39* |  |  |  |  |
| *HOMER2* |  | x |  |  |  |
| *HSD17B4* |  |  |  | Perrault s. |  |
| *ILDR1* | *DFNB42* |  |  |  |  |
| *KARS* | *DFNB89* |  |  |  |  |
| *KCNE1* |  |  |  | *JLNS2* |  |
| *KCNJ10* |  |  |  | *SeSAMES* |  |
| *KCNQ1* |  |  |  | *JLNS1* |  |
| *KCNQ4* |  | *DFNA2A* |  |  |  |
| *KITLG* |  | *DFNA69* |  |  |  |
| *LARS2* |  |  |  | Perrault s. |  |
| *LHFPL5* | *DFNB66/67* |  |  |  |  |
| *LOXHD1* | *DFNB77* |  |  |  |  |
| *LRTOMT* | *DFNB63* |  |  |  |  |
| *MARVELD2* | *DFNB49* |  |  |  |  |
| *MIR96* |  | *DFNA50* |  |  |  |
| *MSRB3* | *DFNB74* |  |  |  |  |
| *MYH14* |  | *DFNA4* |  |  |  |
| *MYH9* |  | *DFNA17* |  |  |  |
| *MYO15A* | *DFNB3* |  |  |  |  |
| *MYO3A* | *DFNB30* |  |  |  |  |
| *MYO6* | *DFNB37* | *DFNA22* |  |  |  |
| *MYO7A* | *DFNB2* | *DFNA11* |  | *USH1B* |  |
| *OSBPL2* | *DFNA67* |  |  |  |  |
| *OTOA* | *DFNB22* |  |  |  |  |
| *OTOF* | *DFNB9* |  |  |  |  |
| *OTOG* | *DFNB18B* |  |  |  |  |
| *OTOGL* | *DFNB84* |  |  |  |  |
| *P2RX2* | *DFNA41* |  |  |  |  |
| *PCDH15* | *DFNB23* |  |  | *USH1F* |  |
| *PDZD7* | x |  |  | *USH2* (digenic with *ADGRV1*) | *USH2A* |
| *PEX1* |  |  |  | PBD |  |
| *PEX2* |  |  |  | PBD |  |
| *PEX3* |  |  |  | PBD |  |
| *PEX5* |  |  |  | PBD |  |
| *PEX6* |  |  |  | PBD |  |
| *PEX7* |  |  |  | PBD |  |
| *PEX10* |  |  |  | PBD |  |
| *PEX12* |  |  |  | PBD |  |
| *PEX13* |  |  |  | PBD |  |
| *PEX14* |  |  |  | PBD |  |
| *PEX16* |  |  |  | PBD |  |
| *PEX19* |  |  |  | PBD |  |
| *PEX26* |  |  |  | PBD |  |
| *PHYH* |  |  |  | PBD |  |
| *PNPT1* | *DFNB70* |  |  |  |  |
| *POU3F4* |  |  | *DFNX2* |  |  |
| *POU4F3* |  | *DFNA15* |  |  |  |
| *PRPS1* |  |  | *DFNX1* |  |  |
| *PTPRQ* | *DFNB84* |  |  |  |  |
| *RDX* | *DFNB24* |  |  |  |  |
| *SERPINB6* | *DFNB91* |  |  |  |  |
| *SIX1* |  | *DFNA23* |  |  |  |
| *SLC17A8* |  | *DFNA25* |  |  |  |
| *SLC26A4* | *DFNB4* |  |  | *PDS* |  |
| *SLC26A5* | *DFNB61* |  |  |  |  |
| *SYNE4* | *DFNB76* |  |  |  |  |
| *TBC1D24* | *DFNB86* | *DFNA65* |  |  |  |
| *TECTA* | *DFNB21* | *DFNA8/12* |  |  |  |
| *TIMM8A* |  |  | *DDS* |  |  |
| *TJP2* |  | *DFNA51* |  |  |  |
| *TMC1* | *DFNB7/11* | *DFNA36* |  |  |  |
| *TMEM132E* | *DFNB99* |  |  |  |  |
| *TMIE* | *DFNB6* |  |  |  |  |
| *TMPRSS3* | *DFNB8/10* |  |  |  |  |
| *TNC* |  | *DFNA56* |  |  |  |
| *TPRN* | *DFNB79* |  |  |  |  |
| *TRIOBP* | *DFNB28* |  |  |  |  |
| *TSPEAR* | *DFNB98* |  |  |  |  |
| *USH1C* | *DFNB18* |  |  | *USH1C* |  |
| *USH1G* |  |  |  | *USH1G* |  |
| *USH2A* |  |  |  | *USH2A* |  |
| *WFS1* |  | *DFNA6* |  |  |  |
| *WHRN* | *DFNB31* |  |  | *USH2D* |  |
| **n = 119** |  |  |  |  |  |
|  |  |  |  |  |  |

**TABLE S2**

**Retinal dystrophy genes analyzed by NGS in this study.** ADRP, autosomal dominant retinitis pigmentosa; ARRP, autosomal recessive retinitis pigmentosa; CD/COD, cone dystrophy; CORD/CRD, cone-rod dystrophy; MD, macular dystrophy; LCA, Leber congenital amaurosis; CSNB, congenital stationary night blindness. Where designations indicate loci, they are written in italics.

ABL, abetalipoproteinemia; ACHM, achromatopsia; ADNIV, dominant neoovascular inflammatory vitreoretinopathy; ALMS, Alström syndrome; BBS, Bardet-Biedl syndrome; BCD, Bietti crystalline corneoretinal dystrophy; BNHS, Boucher-Neuhauser syndrome; CLN, ceroidlipofuscinosis; deg., degeneration; ESC, recessive enhanced S-cone syndrome; JBTS, Joubert syndrome; JS, Jeune syndrome; MCDR, macular dystrophy, retinal; MCPH, microcephaly; MDPT, macular dystrophy, patterned; MSS, Mainzer Saldino syndrome; NCMD, North Carolina macular dystrophy; PHARC, polyneuropathy, hearing loss, ataxia, retinitis pigmentosa, and cataract; PPCRA, pigmented paravenous chorioretinal atrophy; PTBHS, Poretti-Boltshauser syndrome; RCD, retinal cone dystrophy; RD, retinal degeneration; SCRA, Sveinsson chorioretinal atrophy; SFD, Sorsby's fundus dystrophy; SLSN, Senior Loken syndrome; STGD, Stargardt disease; VMD, vitelliform macular dystrophy; (?), disease association questionable.

| **Gene** | **ADRP** | **ARRP** | | **CD, COD, CRD, MD** | **LCA** | | **CSNB** | | **Other** | |
| --- | --- | --- | --- | --- | --- | --- | --- | --- | --- | --- |
| *ABCA4* |  | *RP19* | | *CORD3* (ar), *STGD1* (ar) |  | |  | |  | |
| *ABHD12* |  | x | |  |  | |  | | PHARC (ar) | |
| *ACBD5* |  |  | | CRD  (ar,+psychomotor delay) |  | |  | |  | |
| *ADAM9* |  |  | | *CORD9* (ar) |  | |  | |  | |
| *ADAMTS18* |  |  | |  | x (ar) | |  | |  | |
| *ADIPOR1* |  | x | |  |  | |  | |  | |
| *AGBL5* |  | *RP75* | |  |  | |  | |  | |
| *AIPL1* |  |  | | CRD (ad) | *LCA4* | |  | |  | |
| *ALMS1* |  |  | |  | x (ar) | |  | | ALMS (ar) | |
| *ARL2BP* |  | x | |  |  | |  | |  | |
| *ARL6* |  | *RP55* | |  |  | |  | | *BBS3* (ar) | |
| *ASRGL1* |  | x | |  |  | |  | |  | |
| *BBS2* |  | *RP74* | |  |  | |  | | *BBS2* (ar) | |
| *BBS4* |  |  | |  |  | |  | | *BBS4* (ar) | |
| *BEST1* | *RP50* | x | | CRD (ar,ad) | x (ar) | |  | |  | |
| *C1QTNF5* |  |  | | MD (ad) |  | |  | |  | |
| *C2orf71* |  | *RP54* | |  |  | |  | |  | |
| *C21orf2* |  |  | | CRD (ar) |  | |  | | JS (ar) | |
| *C8orf37* |  | *RP64* | | *CORD16* (ar) |  | |  | | *BBS21* (ar) | |
| *CA4* | *RP17* |  | |  |  | |  | |  | |
| *CABP4* |  |  | | *CRD* (ar) |  | | *CSNB2B* | |  | |
| *CACNA1F* |  |  | | *CORDX3* (X-linked) |  | | *CSNB2*, *CSNB2A*,  *CSNBX2* (X-linked) | |  | |
| *CACNA2D4* |  |  | | *RCD4* (ar) |  | |  | |  | |
| *CAPN5* |  |  | |  |  | |  | | ADNIV (ad) | |
| *CC2D2A* |  |  | |  |  | |  | | *JBTS9* (ar) | |
| *CDH3* |  |  | | MD (ar,+hypotrichosis) |  | |  | |  | |
| *CDH16* |  | x | |  |  | |  | |  | |
| *CDHR1* |  | *RP65* (ar) | | *CORD15* (ar) |  | |  | |  | |
| *CEP290* |  |  | |  | *LCA10* | |  | | *BBS14*, *JBTS5*, *SLSN6* (ar) | |
| *CERKL* |  | *RP26* | | ar |  | |  | |  | |
| *CLRN1* |  | *RP61* | |  |  | |  | | *USH3A* (ar) | |
| *CNGA1* |  | *RP49* | |  |  | |  | |  | |
| *CNGA3* |  |  | | CRD |  | |  | | *ACHM2* (ar) | |
| *CNGB1* |  | *RP45* | |  |  | |  | |  | |
| *CNGB3* |  |  | | CD (ar) |  | |  | | *ACHM3* (ar) | |
| *CNNM4* |  |  | | CRD (ar,+amelogenesis imperfecta) |  | |  | |  | |
| *CRB1* |  | *RP12* | |  | *LCA8* (ar) | |  | | PPCRA | |
| *CRX* | x |  | | *CRD2* (ad) | *LCA7* (ar,ad) | |  | |  | |
| *CTNNA1* |  |  | | *MDPT2* (ad) |  | |  | |  | |
| *CYP4V2* |  | x | |  |  | |  | | BCD (ar) | |
| *DHDDS* |  | *RP59* | |  |  | |  | |  | |
| *DHX38* |  | x | |  |  | |  | |  | |
| *DTHD1* |  |  | |  | x (ar,+myopathy) | |  | |  | |
| *ELOVL4* |  |  | | *STGD3* (ad) |  | |  | |  | |
| *EMC1* |  | x | |  |  | |  | |  | |
| *EYS* |  | *RP25* | |  |  | |  | |  | |
| *FAM161A* |  | *RP28* | |  |  | |  | |  | |
| *FLVCR1* |  | x (+posterior column ataxia) | |  |  | |  | |  | |
| *GDF6* |  |  | |  | *LCA17* (ar) | |  | |  | |
| *GNAT1* |  |  | |  |  | | *CSNBAD3* (ad)  CSNB (ar) | |  | |
| *GNPTG* |  | x (+skeletal abnormalities) | |  |  | |  | |  | |
| *GPR179* |  |  | |  |  | | *CSNB1E* (ar) | |  | |
| *GRID2* |  | x (+ataxia) | |  |  | |  | |  | |
| *GRK1* |  |  | |  |  | | CSNB (ar) | |  | |
| *GRM6* |  |  | |  |  | | *CSNB1B* (ar) | |  | |
| *GUCA1A* |  |  | | *COD3*, *CORD14* (ad) |  | |  | |  | |
| *GUCA1B* | *RP48* |  | | ad |  | |  | |  | |
| *GUCY2D* |  |  | | *CORD6* (ad) | *LCA1* (ar) | |  | |  | |
| *HGSNAT* |  | *RP73* | |  |  | |  | |  | |
| *HK1* | x |  | |  |  | |  | |  | |
| *IDH3B* |  | *RP46* | |  |  | |  | |  | |
| *IFT140* |  | x | | x |  | |  | | MSS (ar) | |
| *IFT172* |  | *RP71* | |  |  | |  | | BBS (ar) | |
| *IMPDH1* | *RP10* |  | |  | *LCA11* (ad) | |  | |  | |
| *IMPG2* |  | *RP56* | |  |  | |  | |  | |
| *IQCB1* |  |  | |  | x (ar) | |  | | *SLSN5* | |
| *ITM2B* | x |  | |  |  | |  | |  | |
| *KCNJ13* |  |  | |  | *LCA16* (ar) | |  | | vitreoretinal deg. (ad) | |
| *KCNV2* |  |  | | *RCD3B* (ar) |  | |  | |  | |
| *KIAA1549* |  | x | |  |  | |  | |  | |
| *KIZ* |  | *RP69* | |  |  | |  | |  | |
| *KLHL7* | *RP42* |  | |  |  | |  | |  | |
| *LAMA1* |  |  | |  |  | |  | | PTBHS (ar) | |
| *LCA5* |  |  | |  | *LCA5* (ar) | |  | |  | |
| *LRAT* |  | x | |  | *LCA14* (ar) | |  | |  | |
| *LRIT3* |  |  | |  |  | | CSNB (ar) | |  | |
| *MAK* |  | *RP62* | |  |  | |  | |  | |
| *MERTK* |  | *RP38* | |  |  | |  | |  | |
| *MFSD8* |  |  | | MD (ar) |  | |  | | *CLN7* (ar) | |
| *MIR204* | x (+coloboma) |  | |  |  | |  | |  | |
| *MTTP* |  |  | |  |  | |  | | ABL (ar) | |
| *MVK* |  | x | |  |  | |  | |  | |
| *NEK2* |  | *RP67* | |  |  | |  | |  | |
| *NEUROD1* |  | x | |  |  | |  | |  | |
| *NMNAT1* |  |  | |  | *LCA9* (ar) | |  | |  | |
| *NR2E3* | *RP37* | *RP37* | | ESC (ar) |  | |  | |  | |
| *NRL* | *RP27* | x | |  |  | |  | |  | |
| *NYX* |  |  | |  |  | | *CSNB1A*  *CSNB4* (ar) | |  | |
| *OR2W3* | x (?) |  | |  |  | |  | |  | |
| *OTX2* |  |  | |  | x (ad,+pituitary dysfunction) | |  | | pattern dystrophy (ad) | |
| *PCYT1A* |  |  | | CRD (ar,+skeletal disease) |  | |  | |  | |
| *PDE6A* |  | *RP43* | |  |  | |  | |  | |
| *PDE6B* |  | *RP40* | |  |  | | *CSNB3*  *CSNBAD2* (ad) | |  | |
| *PDE6C* |  |  | | *COD4* (ar) |  | |  | | *ACHM5* (ar) | |
| *PDE6G* |  | *RP57* | |  |  | |  | |  | |
| *PITPNM3* |  |  | | *CORD5* (ad) |  | |  | |  | |
| *PLA2G5* |  |  | |  |  | |  | | benign fleck retina (ar) | |
| *PLK4* |  |  | |  |  | |  | | RD+MCPH,↓growth (ar) | |
| *PNPLA6* |  |  | |  |  | |  | | BNHS (ar) | |
| *POC1B* |  |  | | *CORD20* (ar) |  | |  | | JBTS | |
| *PRCD* |  | *RP36* | |  |  | |  | |  | |
| *PRDM13* |  |  | | NCMD (ad) |  | |  | |  | |
| *PROM1* |  | *RP41* | | *STGD4*, *CORD12*,  *MCDR2* (all ad) |  | |  | |  | |
| *PRPF3* | *RP18* |  | |  |  | |  | |  | |
| *PRPF31* | *RP11* |  | |  |  | |  | |  | |
| *PRPF4* | *RP70* |  | |  |  | |  | |  | |
| *PRPF6* | *RP60* |  | |  |  | |  | |  | |
| *PRPF8* | *RP13* |  | |  |  | |  | |  | |
| *PRPH2* | *RP7* | digenic (with *ROM1*) | | *MDPT1* (ad), *VMD3* (ad) | *LCA18* (ar,ad) | |  | |  | |
| *RAB28* |  |  | | *CORD18* (ar) |  | |  | |  | |
| *RAX2* |  |  | | *CORD11* (ad) |  | |  | |  | |
| *RBP3* |  | *RP66* | | *MCDR1* (ad) |  | |  | |  | |
| *RBP4* |  |  | |  |  | |  | | RPE degeneration (ar) | |
| *RD3* |  |  | |  | *LCA12* (ar) | |  | |  | |
| *RDH5* |  | fundus albipunctatus (ar) | | CD (ar) |  | |  | |  | |
| *RDH11* |  | x | |  |  | |  | |  | |
| *RDH12* | *RP53* |  | |  | *LCA13* | |  | |  | |
| *RGR* |  | *RP44* | |  |  | |  | | choroidal sclerosis (ad) | |
| *RGS9* |  |  | | delayed cone adapt. (ar) |  | |  | |  | |
| *RGS9BP* |  |  | | delayed cone adapt. (ar) |  | |  | |  | |
| *RHBDD2* |  | x | |  |  | |  | |  | |
| *RHO* | *RP4* | *RP4* | |  |  | | *CSNBAD1* (ad) | |  | |
| *RIMS1* |  |  | | *CORD7* (ad) |  | |  | |  | |
| *RLBP1* |  | x | |  |  | |  | |  | |
| *ROM1* | x | digenic (with *PRPH2*) | |  |  | |  | |  | |
| *RP1* | *RP1* (?) | x | |  |  | |  | |  | |
| *RP1L1* |  |  | | MD (ad) |  | |  | |  | |
| *RPE65* | x | *RP20* | |  | *LCA2* | |  | |  | |
| *RPGR* |  |  | | CD, MD (X-linked) |  | |  | | RP (X-linked) | |
| *RPGRIP1* |  |  | | *CORD13* (ar) | *LCA6* (ar) | |  | |  | |
| *SAG* |  | *RP47* | |  |  | |  | |  | |
| *SEMA4A* | *RP35* |  | | *CORD10* (ad) |  | |  | |  | |
| *SLC7A14* |  | *RP68* | |  |  | |  | |  | |
| *SLC24A1* |  | x (ar) | |  |  | | *CSNB1D* (ar) | |  | |
| *SNRNP200* | *RP33* |  | |  |  | |  | |  | |
| *SPATA7* |  |  | |  | *LCA3* | |  | |  | |
| *SPP2* | x |  | |  |  | |  | |  | |
| *TEAD1* |  |  | |  |  | |  | | SCRA (ad) | |
| *TIMP3* |  |  | | SFD (ad) |  | |  | |  | |
| *TOPORS* | *RP31* |  | |  |  | |  | |  | |
| *TRNT1* |  | x | |  |  | |  | |  | |
| *TRPM1* |  |  | |  |  | | *CSNB1C* (ar) | |  | |
| *TTC8* |  | *RP51* | |  |  | |  | | *BBS8* | |
| *TTPA* |  | x (+ataxia) | |  |  | |  | |  | |
| *TTLL5* |  |  | | *CORD19* (ar) |  | |  | |  | |
| *TUB* |  | x (+obesity) | |  |  | |  | |  | |
| *TUBGCP4* |  |  | |  |  | |  | | RD+MCPH,↓growth (ar) | |
| *TUBGCP6* |  |  | |  |  | |  | | RD+MCPH (ar) | |
| *TULP1* |  | *RP14* | |  | *LCA15* | |  | |  | |
| *UNC119* |  |  | | CRD (ad) |  | |  | |  | |
| *USH1C* |  | x | |  |  | |  | | *USH1C* | |
| *USH2A* |  | *RP39* | |  |  | |  | | *USH2A* | |
| *WDR19* |  |  | |  |  | |  | | JS (ar) | |
| *ZNF408* |  | *RP72* (+vitreal alterations) | |  |  | |  | |  | |
| *ZNF513* |  | *RP58* | |  |  | |  | |  | |
| **n = 155** |  |  | |  |  | |  | |  | |
|  |  |  |  | | |  | |  | |  |
